# Self-Perception of High School Preparation and Readiness for Pharmacy Education

**DOI:** 10.3390/pharmacy14030080

**Published:** 2026-05-30

**Authors:** Shantanu Rao, Kimberly A. Pesaturo, Jennifer Grundey, Timothy Burkart, Devyn Warnement

**Affiliations:** 1 College of Pharmacy, The University of Findlay, Findlay, OH 45840, USA; burkart@findlay.edu (T.B.); devynjess@gmail.com (D.W.); 2 College of Pharmacy and Health Sciences, Western New England University, Springfield, MA 01119, USA; kimberly.pesaturo@wne.edu; 3 College of Pharmacy, Ohio Northern University, Ada, OH 45810, USA; j-kline.3@onu.edu

**Keywords:** high school preparation, student preparedness, pharmacy education, STEM readiness, skill development

## Abstract

Background: The rigor of the Doctor of Pharmacy (PharmD) curricula is reflected in its math- and science-heavy prerequisite requirements. As such, it is critical to evaluate whether students perceive high school coursework and experiences as adequate preparation for PharmD programs. Methods: Pre-professional and professional PharmD students from three institutions completed a survey assessing satisfaction with high school preparation, including math and science education, skill development, and exposure to pharmacy-related experiences. Results: A total of 148 responses were analyzed; 72% identified as female and 81% as White (non-Hispanic/Latino). Most respondents reported satisfaction with their high school math (80.4%) and science (82.4%) education, and 73.7% felt academically prepared for pharmacy school. Additionally, 79.8% were satisfied with skills gained in high school. Experiences such as attending pharmacy-related events (81.1%), contact with healthcare professionals (87.8%), and prior employment (85.2%) were widely viewed as beneficial. Students highlighted communication, problem-solving, and time management as key skills developed. However, gaps were noted in study strategies, presentation abilities, and time management. Conclusions: Students generally felt well-prepared academically and skill-wise for PharmD programs. Expanding pharmacy-related exposure and targeted skill development opportunities in high school may further enhance readiness for future pharmacy students.

## 1. Introduction

Doctor of Pharmacy (PharmD) programs in the United States (US) have recently experienced decreases in student enrollment coupled with an increased attrition rate [1]. In 2023, PharmD student enrollments were down 3% compared to the prior year, and attrition was estimated at an overall 13.4% from 2018 to 2023. The US Bureau of Labor Statistics estimates that pharmacist employment will grow by 5% from 2024 to 2034, which implies a disparity between pharmacists entering the workforce and workforce needs [2]. To meet anticipated future demands, it is crucial that students entering PharmD programs are sufficiently prepared for rigorous programmatic content and possess necessary transferable skills to maximize graduation rates and minimize attrition. Therefore, the high-school-to-PharmD pipeline is one area that may require support to enhance student, and thereby professional, outcomes.

The rigorous PharmD curriculum is reflected in its prerequisite requirements, which emphasize math and science coursework [3]. This aligns with the PharmD degree’s required curricular elements in biomedical, clinical, pharmaceutical, and social and administrative sciences [4]. Further, strong preparation in science, technology, engineering, and math coursework have been associated with later career success [5]. Despite math and science serving as strong foundations for the PharmD program, concerns have been identified in areas of high school science, technology, engineering, and math (STEM) performance, including gender gaps in course selection [6]. Decreasing numbers of high school STEM teachers and perceived STEM teacher qualifications have been observed [7], implying that the overall quality of high school STEM education, and thus student preparation, may be reduced.

To counter potential educational gaps, several studies have examined factors that may impact student perceptions of post-secondary STEM and health career education. For example, outreach programs have been shown to enhance high school student interest in post-secondary medical education and career pursuance [8,9,10]. Anderson and colleagues showed that being encouraged to pursue a PharmD degree, having positive work or volunteer experience, or, to a lesser extent, experiencing “career days,” all potentially influenced student career decisions [11]. Longer-exposure programs, such as summer pharmacy camps or youth science programs, have additionally been shown to maintain high school student interest in pharmacy and health professions [12,13]. Overall, having a high awareness of one’s future field may result in a higher interest level in pursuing that field [14].

Beyond STEM preparation and career exposure, the PharmD career pathway has been associated with the need for strong transferable skills, such as communication skills [15]. Communication skills are modifiable, particularly in healthcare education [16], and targeted training in communication may potentially improve associated skills [17]. Ultimately, how high school students perceive their preparation for PharmD education may impact their success. One standard practice of US-based PharmD programs is to enroll in a “0–6” format in which students can complete their pre-professional and professional PharmD coursework within six years following high school graduation. It is typical within this framework that students make decisions related to higher education in their final year of high school, just prior to college entry. Therefore, the purpose of this study was to determine how PharmD students in direct-entry programs perceived their high school didactic, skill-based, and professional exposures affecting their PharmD education. This study sought to examine the self-perception of student preparedness for pharmacy education during high school.

## 2. Materials and Methods

To determine the preparedness of students entering pharmacy school in this pilot study, a questionnaire was designed and distributed (Microsoft^®^ Forms) to pharmacy students at three 0–6 pharmacy programs in the United States: The University of Findlay (UF), Ohio Northern University (ONU), and Western New England University (WNE). Because no existing survey instrument adequately addressed the objectives of our study, a novel questionnaire was developed by the investigators. Item generation was informed by the authors’ educational background, experience working with pre-pharmacy students, exposure to current enrollment practices in respective colleges, thematic issues identified during routine assessment practices, and literature review, analysis, and synthesis [11,18,19]. The draft instrument underwent iterative revision to establish content relevance and clarity before survey deployment. All students in the 0–6 programs were invited to participate, regardless of the current year in their respective program. After sending the survey link, the researchers followed up with periodic reminders (in-person and via email at respective institutions) to promote survey participation. Demographic information was collected to confirm responses were in line with demographic trends of PharmD student cohorts. In addition, this anonymous survey collected information about math and science classes taken by students while in high school. Students’ perceptions about their academic preparation (specifically math and science coursework), skills development, exposure to pharmacy-related events, and work experience were also collected through the survey. The study’s primary objective was to determine self-perception of academic preparation as it relates to readiness for pharmacy school; secondary objectives were to characterize self-perceptions of skills development, exposure to pharmacy events, and work experience. Survey data was collected between September 2024 and November 2025 across the three institutions. The complete survey questionnaire is offered as a supplemental resource (Supplementary Materials). Descriptive analysis of survey data was conducted using Microsoft^®^ (Redmond, WA, USA) Excel. Responses for ‘select all that apply’ questions were also coded using Microsoft^®^ Excel (Redmond, WA, USA) and key themes were tabulated. Data collected for our primary objective was stratified to analyze major inter-institutional differences, if any, while data pertaining to our secondary objectives was aggregated to determine overall trends among pharmacy students. The research design was reviewed and deemed exempt by the University of Findlay’s Institutional Review Board (#1855).

## 3. Results

A total of 148 responses (20.3%) were received across three institutions. Institutional response rates were found to be comparable: 17% at ONU (n = 71), 34% at UF (n = 43), and 20% (n = 34) at WNE. Across the three institutions, students from all academic years participated in our survey: 25% in pre-professional year 1, 13% in pre-professional year 2, 17% in professional year 1, 18% in professional year 2, 18% in professional year 3, and professional year 4. About 72% of survey participants were female and 81% of respondents were White (not Hispanic or Latino) compared with current national demographic data indicating 67.8% of students enrolled in PharmD programs were women and 21.6% were underrepresented minority students [1]. Student perception of high school preparedness was determined via several survey questions ascertaining level of satisfaction or perceived preparedness prior to joining each respective pharmacy program. Overall, students were extremely satisfied or satisfied with their math (80.4%) and science (82.4%) education in high school (Table 1). Among institutions, this level of extreme satisfaction or satisfaction was found to be comparable for both math (81.7%, 81.4%, and 76.5%) and science education (77.5%, 86.1%, and 88.3%). The aggregate perception of academic preparedness in high school was reported at 73.7% (extremely prepared or prepared). Across institutions, more than two-thirds of participants rated their perception of high school preparation as extremely prepared or prepared (77.5%, 65.1%, and 76.5%). Likewise, the aggregate student perception about their professional skill development in high school was positive, with 79.8% respondents rating it as either satisfied or extremely satisfied and some inter-institutional variation in students’ perception (81.6%, 83.7%, and 70.6%).

In this survey, participants identified specific math and science courses that helped them prepare for the pharmacy curriculum. As shown in Table 2, a majority of the survey participants identified high school precalculus (75.7%) as a class that helped them prepare for pharmacy school. Advanced Placement (AP) calculus (32.4%) and high school calculus (25.7%) were also identified helpful by some participants. Similarly, high school biology (89.9%) and high school chemistry (85.8%) emerged as self-identified science classes that were perceived to improve student preparedness for pharmacy school. High school anatomy and physiology (40.5%) and high school physics (35.1%) were additionally noted to improve perceived student preparedness for pharmacy school. Finally, some survey participants reported that taking college anatomy and physiology (31.1%), high school anatomy and physiology (27.7%), AP chemistry (24.3%), AP biology (22.3%), and college chemistry (20.3%) during high school would have better prepared them to approach the PharmD curriculum.

As our secondary research objectives, some non-academic factors perceived to promote readiness were also examined through this survey. Approximately 72% of respondents mentioned having a parent/family member in the field of pharmacy or speaking to a pharmacy professional while in high school. While in high school, around 10% of survey respondents admitted to having assistance putting together a schedule for high school to help prepare for entering pharmacy school. Around 81% of survey respondents worked while in high school, and about 21% of them reported working in a pharmacy-related field.

**Figure 1:**
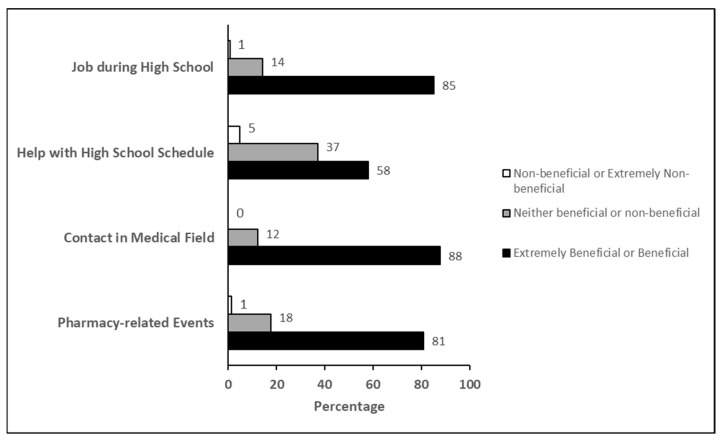
Perceived benefits from non-academic factors in high school. Aggregate values expressed as percentage.

This survey also examined whether participants found such variables to be beneficial in their preparation for college. While in high school (Figure 1), having any type of job experience (85.1%), having assistance putting together a class schedule (58.1%), having a contact in the medical field (87.8%), and attending pharmacy-related events (81.1%) were rated as beneficial or extremely beneficial by our survey respondents when preparing for pharmacy school.

Pharmacy students’ reflection on skill development during high school, as recorded through ‘select all that apply’ questions, is shown in Table 3. Survey participants rated the development of communication skills (73%), problem-solving skills (71.6%), and time-management skills (71.6%) during high school to help them the most with pharmacy education. Development of other skills such as teamwork (64.2%), critical thinking (60.1%), organizational skills (60.1%), and social skills (60.1%) in high school were also identified to be helpful during pharmacy education. At the same time, participants identified the need for improvement in study skills (54.7%), presentation skills (44.6%), and time-management skills (41.2%) in high school prior to enrolling in pharmacy school. Interestingly, while a high proportion of survey respondents mentioned the significance of developing teamwork (60.1%) as a valuable skill for pharmacy school, less than 10% of survey participants wished they would have perfected teamwork during high school. Overall, for skills, no other discernable pattern was identified in the survey results.

## 4. Discussion

This study provides a summary of how pharmacy students perceive their preparedness for a pharmacy curriculum based on many factors and subjects encountered during their high school years. The survey included both academic and non-academic experiences. Respondents overall reported a high or extremely high level of satisfaction with regards to high school preparation in both academic and non-academic activity. In didactic categories, over 80% of respondents surveyed across all three institutions reported satisfaction or extreme satisfaction with both math and science education in high school. Three-quarters of respondents also noted they felt academically prepared for the pharmacy curriculum upon college entry. Direct-entry PharmD programs (0–6) were selected as the target cohort to limit the introduction of recall bias that may occur following elapsed time between high school and a traditional four-year PharmD degree.

Disaggregating the didactic focus yielded more specific insights into courses that respondents perceived as contributing to preparedness. Precalculus, calculus, biology, and chemistry were cited most frequently as being beneficial based on foundations of quantitative skills or life sciences. Advanced courses, particularly those related to life sciences (biology, chemistry, anatomy and physiology) offered at an AP or other college level offering in high school, were also identified as beneficial by respondents. Some students also reported a desire to take these courses but may not have had the opportunity due to individual high school curricular offerings. These observations were unsurprising given the science- and math-centered pharmacy curriculum [3,4]. It is worth noting that the traditional US secondary educational framework, which generally includes grades following elementary (or primary) school through grade 12 (typical completion of high school), allows considerable variability in terms of course pathways and level of rigor. Students may choose to enroll or become placed in high school coursework that can be taken at different levels of rigor, curricula, or depth. One such example included in the scope of this work includes AP and standard-track high school courses. In AP coursework, high school students are generally enrolled in a course that would be considered on par with the increased rigor and depth of a college course, as these courses follow a specific program of study and culminate in an exam that can earn the student college credit [20]. Additionally, early post-secondary (college) curricula often include general education requirements that are scaffolded with previous high school courses, but with greater depth and rigor. Ultimately, there is not one standard high school pathway that precedes college entry, but many iterations that allow varied levels of difficulty and preparation. In addition, high schools vary greatly depending on the school size and its course offerings. While all high schools must meet certain minimum state requirements for graduation, the number of courses offered, the exact content covered and whether advanced, AP or college credit courses are available differs among schools.

Existing literature indicates pre-pharmacy academic preparation, especially in mathematics and the sciences, is a predictor of student success in PharmD programs. Studies have shown that higher pre-pharmacy grade point averages in math and science coursework are associated with improved performance during the PharmD curriculum [21]. Completion of advanced science coursework, such as upper-level biology, has been linked to higher professional GPAs and increased likelihood of on-time PharmD program progression [22,23]. Mathematics preparation also plays a critical role, with stronger prior performance in math associated with success in required PharmD coursework involving pharmaceutical calculations [24,25]. In fact, math assessment scores during student interviews have been identified as a strong indicator for success in pharmacy school [26], further emphasizing the need for adequate high school math preparation. Amongst bridging programs designed to assist student preparedness for PharmD programs, math-based assessment or content were reported to be the most common theme [27]. With the heavy emphasis on math and science courses within the PharmD curricula, it is not surprising that students throughout different cohorts in this pilot study perceive that various math and science courses in high school helped to prepare them for the pharmacy curriculum.

Intentional outreach to high school students can augment interest in pharmacy as a career choice and address the declining enrollment trends across schools of pharmacy. For instance, presentations to high school students provided by current pharmacy students were found to dramatically improve knowledge about the profession [28]. Similarly, week-long or shorter pharmacy programs designed to offer an exploratory experience to high school students have been documented to increase attendees’ knowledge about the profession, expand their interest in the profession of pharmacy, and enhance enrollment numbers for pharmacy programs [12,29,30]. Results from the present study corroborate the perceived benefits of pharmacy-related events when experienced as high school students. It is therefore imperative for pharmacy schools to design targeted outreach for high school students to enhance their knowledge about the profession of pharmacy and in turn increase students’ overall interest in joining the profession. Additionally, implementation of prematriculation programs, designed as STEM refresher courses [31,32], can assist high school students in better adapting to the rigors of pharmacy education and increase their long-term success. Widespread implementation of such bridge gap programs can increase the feasibility of pharmacy education for high schoolers who feel less prepared for science-based courses. Additionally, such measures can address the variability of high school curricular offerings across socioeconomic levels and increase diverse demographical interest towards the profession of pharmacy.

Beyond academic preparedness, this study highlights the importance of non-cognitive skills, such as communication, as essential for the transition from high school to a PharmD program. Essential competency standards for pharmacy education require proficiency in communication, problem solving and teamwork, echoing participants’ perceptions that these non-academic skills were helpful to develop before beginning pharmacy school [4]. Effective communication skills have been found to improve clinical decision-making by student pharmacists [33] and, intuitively, pharmacy programs have adopted techniques such as simulation activities [34] or team-building workshops [35] to enhance students’ communication skills. Additionally, developing time-management skills during high school can have a positive impact during pharmacy school. Studies from across the world have underscored the positive correlation between effective time-management skills and students’ academic performance [36,37]. In fact, even a short, well-thought-out preparatory session prior to matriculation was demonstrated to improve pharmacy student performance [38]. Hence, by integrating rigorous science with early training in professional communication and time management, direct-entry PharmD programs can better equip students for the interdisciplinary and patient-centered demands of current pharmacy practice.

Prior pharmacy work experience has been identified to positively impact student pharmacists. Interestingly, about one-fifth of study participants indicated working in a pharmacy-related job before starting pharmacy school. Past studies have indicated that prior pharmacy work experience may help students to develop professional identity early in their pharmacy school journey [39]. While the quantitative impact of such work experience on a student’s academic performance may be minimal [39,40], exposure to the profession of pharmacy prior to joining pharmacy school may help high school students to become more comfortable with analyzing drug information and professional communication in a pharmacy setting [40].

Overall, there is a distinct lack in the primary literature regarding student perceptions on how their high school education and surrounding factors prepared them for their PharmD program of study. Further, given the variable nature of high school experiences, this work does not address the correlation between high school experiences and the perceptions reported here. Additional limitations may be found in the present study. First, the results of this study are limited to 0–6 year or direct entry programs at participating institutions in the United States and may not be applicable to traditional four-year Doctor of Pharmacy programs. Also, the study population lacks a strong minority cohort, which may also impact the perceptions of access or regional offerings for both standard and advanced math and life science courses in high school curricula. Data related to the participants’ high school type (public or private) and socioeconomic factors were not collected. Thus, the researchers are unable to examine whether responses differ based on educational background or personal or school-related socioeconomic factors. Additional research could aim to incorporate these factors to allow for a more comprehensive analysis. Researchers also recognize that while some standardization exists between courses offered at different high schools (for example, all AP biology courses follow an established curriculum and are designed to prepare students for the standardized AP test), there is still likely great content and quality variability across courses offered in various high schools. Finally, there are likely numerous factors that contribute to students’ real and perceived barriers to success in PharmD programs. While this study focused on student self-reflection, the researchers recognize this is not an objective measure of Doctor of Pharmacy curriculum readiness. However, given the broad and varied nature of US-based secondary educations, the overall lack of consistency with this study’s results are not surprising. Future iterations of similar studies should seek to underscore reproducibility and/or psychometric validation.

## 5. Conclusions

As PharmD programs adjust to the continued decrease in enrollment, colleges have a vested interest in understanding students’ perceptions on what best prepares them for the PharmD curriculum. Starting at the high school level, counselors could encourage prospective pharmacy students to take available math and science courses while in high school and seek employment in the pharmacy or medical field as ways to feel prepared. PharmD programs could seek opportunities to connect prospective students to current medical professionals and offer innovative pharmacy-related events to drive interest in the profession. PharmD programs also might look at ways to model or teach skills students wish they had a better handle on such as studying, presenting, and time management. Further research would be helpful to evaluate whether student perceptions align with actual preparedness as evidenced by PharmD student outcomes.

## Figures and Tables

**Table 1:** Level of satisfaction or preparedness in high school. Values expressed as percentage (n).

Students’ Perspective on Level of Satisfaction or Preparedness						
Satisfaction	Institution	Likert Scale Responses [% (n)]				
		Extremely Satisfied	Satisfied	Neither Satisfied nor Dissatisfied	Dissatisfied	Extremely Dissatisfied
Level of **Satisfaction** with **Math Education**	Institution 1	39.4 (28)	42.3 (30)	8.5 (6)	8.5 (6)	1.4 (1)
	Institution 2	30.2 (13)	51.2 (22)	4.7 (2)	11.6 (5)	2.3 (1)
	Institution 3	26.5 (9)	50 (17)	20.6 (7)	2.9 (1)	0.0 (0)
	**Aggregate**	33.8 (50)	46.6 (69)	10.1 (15)	8.1 (12)	1.4 (2)
Level of **Satisfaction** with **Science Education**	Institution 1	46.5 (33)	31.0 (22)	9.9 (7)	9.9 (7)	2.8 (2)
	Institution 2	23.3 (10)	62.8 (27)	4.7 (2)	9.3 (4)	0.0 (0)
	Institution 3	41.2 (14)	47.1 (16)	5.9 (2)	5.9 (2)	0.0 (0)
	**Aggregate**	38.5 (57)	43.9 (65)	7.4 (11)	8.8 (13)	1.4 (2)
Level of **Satisfaction** with **Skill Development** in High School	Institution 1	23.9 (17)	57.7 (41)	11.3 (8)	7.0 (5)	0.0 (0)
	Institution 2	18.6 (8)	65.1 (28)	9.3 (4)	7.0 (3)	0.0 (0)
	Institution 3	14.7 (5)	55.9 (19)	20.6 (7)	8.8 (3)	0.0 (0)
	**Aggregate**	20.3 (30)	59.5 (88)	12.8 (19)	7.4 (11)	0.0 (0)
**Preparedness**		**Extremely prepared**	**Prepared**	**Neither prepared nor unprepared**	**Unprepared**	**Extremely unprepared**
**Level** of **Academic Preparedness** in High School	Institution 1	25.4 (18)	52.1 (37)	9.9 (7)	8.5 (6)	4.2 (3)
	Institution 2	16.3 (7)	48.8 (21)	20.9 (9)	14.0 (6)	0.0 (0)
	Institution 3	11.8 (4)	64.7 (22)	14.7 (5)	8.8 (3)	0.0 (0)
	**Aggregate**	19.6 (29)	54.1 (80)	14.2 (21)	10.1 (15)	2.0 (3)

**Table 2:** List of high school classes perceived or speculated to improve students’ preparedness for pharmacy schools. Aggregate data across three institutions expressed as percentage of respondents.

Math Classes That Improved Student Preparedness for Pharmacy School		Science Classes That Improved Student Preparedness for Pharmacy School		Math & Science Classes You Wish You Had Taken to Prepare for Pharmacy School	
Class	% (n)	Class	% (n)	Class	% (n)
High School Precalculus	75.7 (112)	High School Biology	89.9 (133)	College Anatomy and Physiology	31.1 (46)
AP Calculus	32.4 (48)	High School Chemistry	85.8 (127)	High school Anatomy and Physiology	27.7 (41)
High School Calculus	25.7 (38)	High School Anatomy and Physiology	40.5 (60)	AP Chemistry	24.3 (36)
College Statistics	20.9 (31)	High school Physics	35.1 (52)	AP Biology	22.3 (33)
High School Statistics	18.9 (28)	AP Chemistry	29.7 (44)	College Chemistry	20.3 (30)
College Calculus	18.9 (28)	AP Biology	28.4 (42)	College Biology	18.2 (27)
College Precalculus	14.9 (22)	College Chemistry	16.2 (24)	College Physics	16.2 (24)
AP Statistics	12.8 (19)	AP Physics	15.5 (23)	None of the Above	15.5 (23)
AP Precalculus	2.7 (4)	College Anatomy and Physiology	14.2 (21)	High School Statistics	12.8 (19)
		College Biology	11.5 (17)	College Calculus	12.8 (19)
		College Physics	11.5 (17)	College Statistics	12.2 (18)
College Credit Plus (CCP) courses: Offered to high school students in which the student must earn a passing grade within the class to receive college credit for the course. Advanced Placement (AP) courses: Offered to high-school students in which the student enrolls in a standardized curriculum that culminates in a national exam which is scored on a scale of 1–5. To receive college credit for the course, the student must meet a predetermined score usually above 3.					

**Table 3:** Summary of participants’ responses about skill development during high school. Aggregate values expressed as percentage of responses amongst student responses.

Developing Which Skills Helped Going into Pharmacy		Skills You Wish You Would Have Perfected Going into Pharmacy	
Skill	%	Skill	%
Communication skills	73.0	Studying skills	54.7
Problem solving	71.6	Presenting skills	44.6
Time management	71.6	Time management	41.2
Teamwork	64.2	Communication skills	30.4
Critical thinking	60.1	Social skills	29.1
Organizational skills	60.1	Leadership	24.3
Social skills	60.1	Adaptability	20.9
Listening	56.8	Critical thinking	20.9
Adaptability	54.1	Customer service skills	20.9
Leadership	52.0	Writing	19.6
Writing	50.7	Organizational skills	16.2
Studying skills	45.9	Problem solving	16.2
Presenting skills	40.5	Listening	12.2
Customer service skills	39.9	Teamwork	9.5

## Data Availability

The original contributions presented in this study are included in the article/Supplementary Materials. Further inquiries can be directed to the corresponding author.

## Supplementary Materials

The following supporting information can be downloaded at: https://www.mdpi.com/article/10.3390/pharmacy14030080/s1, File S1: Survey Questions.
