# Supplementary material for: Self-Perception of High School Preparation and Readiness for Pharmacy Education
Source: Pharmacy (Basel). 2026 May 30;14(3):80. doi: 10.3390/pharmacy14030080 (PMC13307450; doi:10.3390/pharmacy14030080)
Supplement: Supplementary file 1 [file pharmacy-14-00080-s001.zip › pharmacy-4291510-supplementary.pdf]

Supplementary Materials – Survey Questions

Are you under the age of 18?
Yes
No

What is your gender?
Male
Female
Non-binary
Choose not to answer

Please select the ethnicity which most accurately describes how you identify yourself:
American Indian or Alaska Native (Not Hispanic or Latino)
Asian (Not Hispanic or Latino)
Black or African American (Not Hispanic or Latino)
Hispanic or Latino
Native Hawaiian or Other Pacific Islander (Not Hispanic or Latino)
White (Not Hispanic or Latino)
I identify as more than one ethnicity
I do not wish to answer

What year in the pharmacy curriculum are you in?
FY1 (formerly P1)
FY2 (formerly P2)
P1 (formerly P3)
P2 (formerly P4)
P3 (formerly P5)
P4 (formerly P6)

What **math classes** did you take in high school that helped prepare you for the pharmacy curriculum: (Select all that apply)
High school Precalculus
High school Calculus
High school Statistics
AP Statistics
AP Calculus
AP Precalculus
College Calculus
College Statistics
College Precalculus
Other (if selected have a fill in)

What is your level of satisfaction with your **math education** before starting the pharmacy program?
(Extremely Satisfied, Satisfied, Neither Satisfied or Dissatisfied, Dissatisfied, Extremely Dissatisfied)

What **science classes** did you take in high school that helped prepare you for the pharmacy curriculum: (Select all that apply)
High school Chemistry
High school Anatomy and Physiology
High school Biology
High school Physics
AP Chemistry
AP Biology
AP Physics
College Chemistry
College Anatomy and Physiology
College Biology
College Physics
Other (if selection have a fill in)

What is your level of satisfaction with your **science education** before starting the pharmacy program?
(Extremely Satisfied, Satisfied, Neither Satisfied or Dissatisfied, Dissatisfied, Extremely Dissatisfied)

What math or science classes **do you wish you would have taken** during high school to better prepare you for pharmacy education? (Please specify if AP, college, or high school, etc.)
High school Chemistry
High school Anatomy and Physiology
High school Biology
High school Physics
AP Chemistry
AP Biology
AP Physics
College Chemistry
College Anatomy and Physiology
College Biology
College Physics
High school Precalculus
High school Calculus
High school Statistics
AP Statistics
AP Calculus
AP Precalculus
College Calculus
College Statistics
College Precalculus
None of the above
Other (if selection have a fill in)

Did any classes that were not required by the high school curriculum and not previously listed math or science classes help prepare you for college? If so, please list below (ex. medical terminology, pharmacy-related class, etcetera):

Fill in

What high school activities did you participate in that helped prepare you for pharmacy and why do you feel they were beneficial? (ex. Debate club, volunteering, etcetera)
Fill in

Did you feel **well prepared to go into pharmacy school** in terms of academics?
(Extremely prepared, prepared, neither prepared or nor unprepared, Not prepared, extremely not prepared)

Why did you or did you not feel prepared?
Fill in

Did developing any of these **skills** during high school help **prepare you for going into pharmacy**? (select all that apply)
Communication skills
Studying skills
Time management
Teamwork
Critical thinking
Leadership
Problem Solving
Writing
Presenting Skills
Organizational skills
Social Skills
Customer Service Skills
Adaptability
Listening
Other: (list out)

What is your level of satisfaction with your **skills before starting the pharmacy program**?
(Extremely Satisfied, Satisfied, Neither Satisfied or Dissatisfied, Dissatisfied, Extremely Dissatisfied)

What skills **do you wish you would have perfected** before coming to pharmacy school? (Select all that apply).
Communication skills
Studying skills
Time management
Teamwork
Critical thinking
Leadership
Problem Solving
Writing
Presenting Skills
Organizational skills
Social Skills

Customer Service Skills
Adaptability
Listening
None of above
Other: (list out)

Did you **attend/participate** in any of these **pharmacy-related events?** (select all that apply)
Shadowed a pharmacist
Attended a pharmacy summer camp
Spoke with a pharmacist in high school about their career
Spoke with a healthcare worker other than a pharmacist about pharmacy/a medical-related career
Had a school speaker come to speak about pharmacy (possibly a student who went to your high school came back to speak about pharmacy school)
Attended a college-hosted pharmacy event for High Schoolers
Prime the Pump
Did not attend any pharmacy related events
Other (Fill in)

How beneficial is **being exposed to pharmacy-related events** to preparing you for the pharmacy program?
(Extremely beneficial, beneficial, Neither beneficial or non-beneficial, Non-beneficial, Extremely non-beneficial)

Did you have **direct contact** with anyone in the **medical field** (Ex. Having a parent in a medical field, having a family member/friend who is a pharmacy, speaking with someone in a pharmacy, etc.)?
Yes
No

Do you **believe** having <u>contact with someone in the medical field</u> is **beneficial** when choosing pharmacy school?
(Extremely beneficial, beneficial, Neither beneficial or non-beneficial, Non-beneficial, Extremely non-beneficial)

Did you have someone help put together a **schedule** for you in **high school** to help prepare you going into pharmacy school?
Yes
No

If you answered YES to the previous question, who helped put together a schedule for you in high school to help prepare you to go into pharmacy school?
Fill in

How beneficial is **having someone help** put together a high school schedule for preparation for pharmacy school?
(Extremely beneficial, beneficial, Neither beneficial or non-beneficial, Non-beneficial, Extremely non-beneficial)

Did you have a job before entering pharmacy school?
Yes
No

If you answered YES to the previous question, what was your job?
Fill in

How **beneficial** is having a **job** before starting pharmacy school?
(Extremely beneficial, beneficial, Neither beneficial or non-beneficial, Non-beneficial, Extremely non-beneficial)
